# COVID-19 induced psychosis. Should we be concerned?

**DOI:** 10.1192/j.eurpsy.2022.529

**Published:** 2022-09-01

**Authors:** P. Costa, I. Pinto, P. Branco

**Affiliations:** 1 Centro Hospitalar Psiquiátrico de Lisboa, Clínica 2 - Psicogeriatria, Lisboa, Portugal; 2 Centro Hospitalar Psiquiátrico de Lisboa, Clínica 1 - Unidade Partilhada, Lisboa, Portugal

**Keywords:** “COVID-19”, “SARS-CoV-2”, “pandemics”, “psychotic symptoms”, “psychosis”

## Abstract

**Introduction:**

Coronaviruses traditionally are considered to cause pulmonary diseases, often accompanied by gastrointestinal symptoms. Since the COVID-19 pandemic start in early 2020, there have been reports of a high prevalence of neuropsychiatric symptoms. Recent data show significant rates of neuropsychiatric diagnosis over the subsequent 6 months post-infection. Some of the data suggest the COVID-19 as a cause of new-onset psychotic symptoms in patients with no psychiatric history. Delusions, hallucinations, disorganized thoughts, and confusion were the most frequently reported psychotic features which low doses of antipsychotics seem to be helpful.

**Objectives:**

Brief literature review about the relationship between COVID-19 and new-onset psychotic symptomatology.

**Methods:**

Non-systematic review through PubMed research using the terms “COVID-19”, “SARS-CoV-2”, “pandemics”, “psychotic symptoms” and “psychosis”.

**Results:**

The severity of the infection, especially in those with the need for hospitalization/intensive care, seems to have a clear effect on the gravity of subsequent neuropsychiatric symptoms, namely psychosis. Viral invasion of the central neural system, hypercoagulable states, and neuroinflammation are potential associated mechanisms. It’s important to consider the effect of therapies that may have the potential to cause psychosis (eg steroids). According to recent literature, around 0.9-4% of people exposed to the COVID-19 virus develop psychotic episodes, which is much higher than the incidence in the general population.

**Conclusions:**

Post-COVID-19 related psychosis has been reported in different nations. The pathophysiology is yet not clear, although the hyperinflammatory response has been suggested as the main mechanism for the neuropsychiatric manifestations. Given the high number of case reports with similar presentations, it’s important to proceed with more investigations.

**Disclosure:**

No significant relationships.

